# Elastance-derived transpulmonary pressure may overestimate the risk of overdistension in severely obese patients

**DOI:** 10.1186/s13054-023-04453-2

**Published:** 2023-05-04

**Authors:** Samuel Tuffet, Elsa Moncomble, Mohamed Ahmed Boujelben, Anne-Fleur Haudebourg, Armand Mekontso-Dessap, Guillaume Carteaux

**Affiliations:** 1 CHU Henri Mondor-Albert Chenevier, Service de Médecine Intensive Réanimation, Assistance Publique-Hôpitaux de Paris, 51, Avenue du Maréchal de Lattre de Tassigny, 94010 Créteil Cedex, France; 2 Groupe de Recherche Clinique CARMAS, Faculté de Santé, Université Paris Est-Créteil, 94010 Créteil Cedex, France; 3 INSERM U955, Institut Mondor de Recherche Biomédicale, 94010 Créteil Cedex, France

**Keywords:** ARDS, EIT, Transpulmonary pressure, PEEP

Dear editor,

During acute respiratory distress syndrome (ARDS), estimation of transpulmonary pressure (*P*_L_) using esophageal pressure has been proposed to customize the positive end-expiratory pressure (PEEP) in order to avoid alveolar collapse (i.e., positive end-expiratory *P*_L_ [*P*_L, exp_]) while limiting the stress applied to the lung (i.e., limit end-inspiratory *P*_L_ below 20–25 cmH_2_O) [1]. Regarding the latter, the calculation of end-inspiratory *P*_L_ based on the elastance ratio method (*P*_L,end-insp,ER_) is usually preferred because it has been shown to better approximate the *P*_L_ of the non-dependent areas than the direct method (*P*_L,end-insp,direct_) [2]. However, the elastance ratio method relies on the assumption that *P*_L_ is zero at atmospheric pressure, which may be inappropriate in some patients. We herein report such an illustrative case.

A 30-year-old obese woman (BMI 52.5 kg.m^−2^) with past medical history of HIV infection was admitted to the Intensive Care Unit for a moderate ARDS related to undocumented community-acquired pneumonia. After intubation, tidal volume was set at 6.6 mL/kg of predicted bodyweight and respiratory rate at 34 cycles/min. With a PEEP of 6 cmH_2_O, the plateau pressure (*P*_PLAT_) was measured at 30 cmH_2_O. However, an airway opening pressure (AOP) of 16 cmH_2_O [3] was retrieved during low-flow insufflation. Chest CT-scan showed bilateral alveolar consolidations. To customize ventilator’s settings, an esophageal catheter was inserted and electrical impedance tomography (EIT, Enlight 1800, TIMPEL, São Paulo, Brazil) was recorded during decremental PEEP titration from 30 to 6 cmH_2_O, in steps of 2 cmH_2_O, with constant tidal volume and flow rate. Esophageal balloon (Nutrivent, Sidam, Italy) was filled with 4 mL of air. Proper position and filling were verified by chest radiograph, the presence of cardiac artifacts on P_ES_ recording, and a Δ*P*_ES_/Δ*P*_AW_ ratio induced by gentle chest compressions during end-expiratory occlusion at PEEP 18 cmH_2_O at 0.99 [1]. At each step, the following parameters were collected or calculated according to standard formulas: *P*_PLAT_ (measured after 0.5 s end-inspiratory occlusion), total PEEP (PEEP_TOT_, measured after 2 s end-expiratory hold), lung driving pressure (Δ*P*_L_, computed as *P*_L,end-insp,direct_ – *P*_L,exp_) and respiratory system driving pressure (Δ*P*_RS_, defined as *P*_PLAT_ – end-expiratory alveolar pressure, where end-expiratory alveolar pressure was either PEEP_TOT_ or AOP, whichever was greater), lung compliance (*C*_L_) and *C*_RS_, *P*_L, exp_ (defined as end-expiratory alveolar pressure—*P*_es,exp_, where end-expiratory alveolar pressure was either PEEP_TOT_ or AOP, whichever was greater), *P*_L,end-insp,direct_, *P*_L,end-insp,ER_, stress index, and the percentage of collapse and overdistension estimated by EIT.

During the decremental PEEP titration, *P*_PLAT_, *P*_L, exp_, *P*_L, end-insp, direct_, *P*_L, end-insp, ER_, and the percentage of overdistension decreased, while the percentage of collapse increased. Consequently, both Δ*P*_RS_ and Δ*P*_L_ adopted a U-shaped pattern, with a nadir corresponding to a PEEP of 24 cmH_2_O. No significant hemodynamic changes were observed.

The *P*_L, exp_ measurement and EIT assessment of the percentage of collapse resulted in a congruent estimate of the minimum PEEP required to minimize derecruitment: the lowest PEEP associated with positive *P*_L, exp_ corresponded to the lowest PEEP associated with a percentage of collapse < 1% (Fig. 1).

**Fig. 1:**
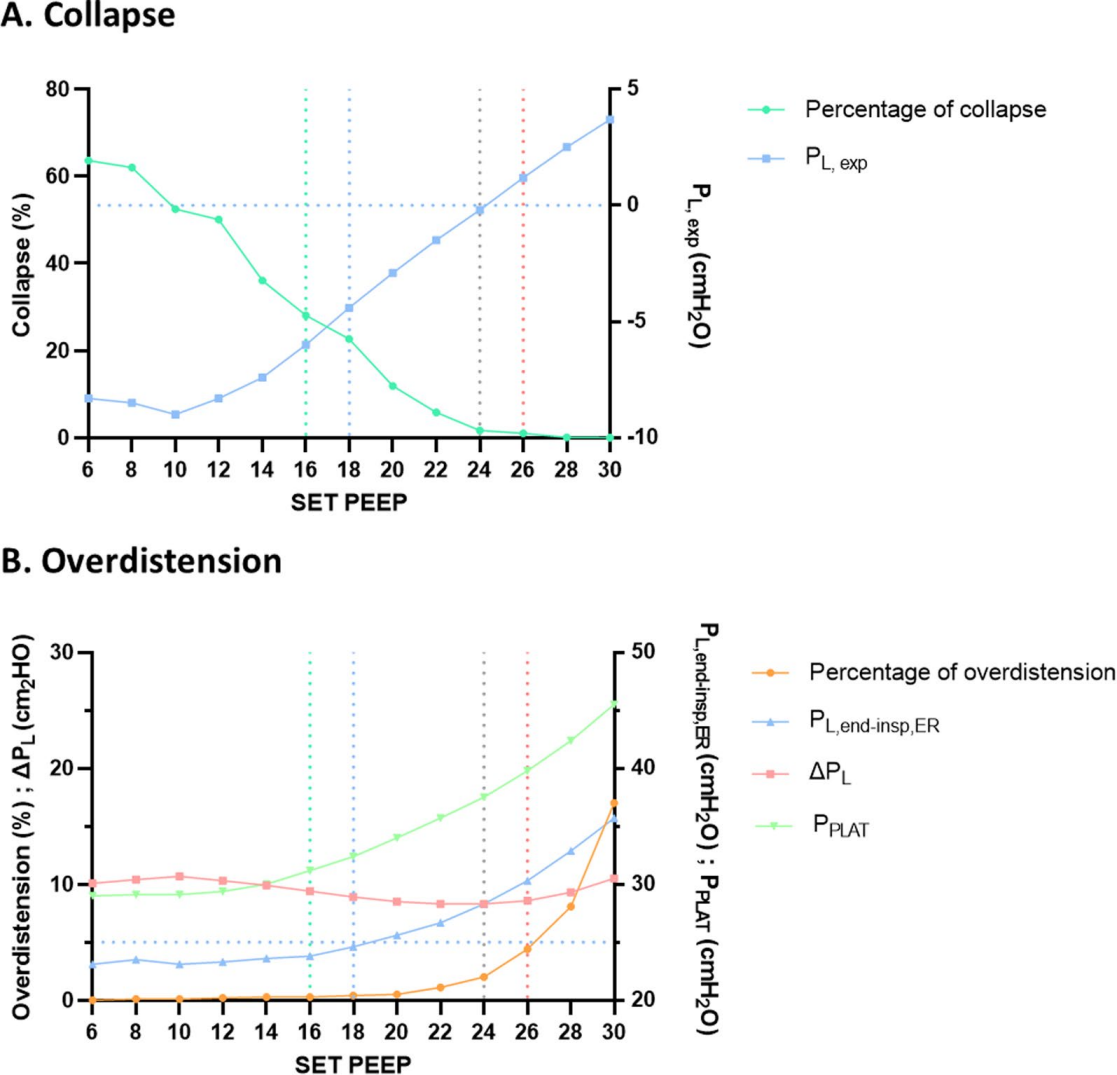
Results of the PEEP titration. **A** collapse indices. Left *Y* axis, green curve: collapse determined by EIT. Right *Y* axis, blue curve: end-expiratory transpulmonary pressure. Blue-dashed horizontal line indicates 0 cmH_2_O. **B** Overdistension indices. Left *Y* axis, orange curve: overdistension determined by EIT. Left *Y* axis, red curve: transpulmonary driving pressure (Δ*P*_L_). Right *Y* axis, blue curve: end-inspiratory, elastance derived transpulmonary pressure (*P*_L, end-insp, ER_); Right *Y* axis, green curve: plateau pressure (*P*_PLAT_). Blue horizontal-dashed line indicates 25 cmH_2_O. On each graph, green vertical-dashed line indicates airway opening pressure; blue vertical-dashed line indicates the highest set PEEP with elastance derived transpulmonary pressure < 25 cmH_2_O; grey vertical-dashed line indicates set PEEP with lowest transpulmonary driving pressure; red vertical-dashed line indicates the lowest level of set PEEP associated with positive end-expiratory transpulmonary pressure, and the lowest level of set PEEP associated with minimal collapse by EIT (defined as collapse < 1%)

In contrast, *P*_L, end-insp, ER_ appeared to overestimate the risk of overdistension, according to EIT assessment: P_L, end-insp, ER_ reached the upper limit of 20–25 cmH_2_O for a PEEP of 6–18 cmH_2_O, respectively. However, between 18 and 24 cmH_2_O, we found no other marker of overdistension: Δ*P*_RS_ and Δ*P*_L_ decreased continuously, the stress index remained below 1, and overdistension measured by EIT was kept negligible (Fig. 1). Conversely, *P*_L, end-insp, direct_ appeared to underestimate the risk of overdistension. In fact, as Δ*P*_L_ increased between PEEP 24 and 30 cmH_2_O and EIT revealed significant overdistension at PEEP 30 cmH_2_O (17%), *P*_L, end-insp, direct_ increased from eight to 14 cmH_2_O between these two levels of PEEP, suggesting a safe range.

Based on this overall assessment, the PEEP was adjusted to 24 cmH_2_O to mitigate the risk of both collapse and overdistension. Two prone sessions were performed and the patient was weaned from mechanical ventilation on day-16.

We herein report a case illustrating that estimating end-inspiratory *P*_L_ using esophageal pressure may significantly misjudge the risk of overdistension in obese patients. Two non-mutually exclusive explanations could account for the discrepancy between *P*_L, end-insp, ER_ and overdistension estimated by EIT in this patient. Firstly, since EIT estimates distension and collapse from the relative change in pixel-level compliance calculated from pixel intratidal variation of impedance and driving pressure, the percentage of overdistension can be altered when the PEEP is set below AOP. In the current case, overdistension estimated by EIT started several levels of PEEP above AOP, lending support to the second explanation, which is that obesity is associated with significantly higher end-expiratory pleural pressure [4], a situation where the conditions for *P*_L, end-insp, ER_ interpretation are no longer met [5]. In that case, EIT assessment may help adjusting PEEP to limit both derecruitment and overdistension. Further studies are needed to determine the consistency of such an observation.

## Data Availability

The datasets used and/or analyzed during the current study are available from the corresponding author on reasonable request.

## Abbreviations

ARDS: Acute respiratory distress syndrome
EIT: Electrical impedance tomography
PEEP: Positive end-expiratory pressure
